# Maternal serum Vitamin B12 and offspring attention-deficit/hyperactivity disorder (ADHD)

**DOI:** 10.1007/s00787-020-01621-5

**Published:** 2020-09-04

**Authors:** Andre Sourander, Sanju Silwal, Subina Upadhyaya, Heljä-Marja Surcel, Susanna Hinkka-Yli-Salomäki, Ian W. McKeague, Keely Cheslack-Postava, Alan S. Brown

**Affiliations:** 1 Department of Child Psychiatry, Research Centre for Child Psychiatry, Institute of Clinical Medicine, Faculty of Medicine, University of Turku, Lemminkäisenkatu 3/Teutori (3rd floor), 20014 Turku, Finland; 2 Department of Child Psychiatry, Turku University Hospital, Turku, Finland; 3 INVEST Research Flagship, University of Turku, Turku, Finland; 4 Faculty of Medicine, University of Oulu, Oulu, Finland; 5 Biobank Borealis of Northern Finland, Oulu, Finland; 6 Department of Biostatistics, Columbia University Mailman School of Public Health, New York, NY, USA; 7 Department of Psychiatry, New York State Psychiatric Institute, Columbia University Irving Medical Center, New York, NY, USA; 8 Department of Epidemiology, Columbia University Mailman School of Public Health, New York, NY, USA

**Keywords:** Attention-deficit/hyperactivity disorder, ADHD, Maternal, Prenatal, Vitamin B12

## Abstract

Maternal Vitamin B12 deficiency during pregnancy is associated with offspring neuropsychiatric disorders. Few previous studies examining this association with attention-deficit/hyperactivity disorder (ADHD) report inconsistent findings. The study examines the association between maternal serum Vitamin B12 levels and offsprings’ risk of ADHD. This study is based on the Finnish Prenatal Study of ADHD with a nested case–control design. All the singleton children born in Finland between January 1998 and December 1999 and diagnosed with ADHD were included in the study. A total of 1026 cases were matched with an equal number of controls on sex, date of birth and place of birth. Maternal Vitamin B12 levels were assessed using a chemiluminescence microparticle immunoassay and archived from maternal serum banks, collected during the first and early second trimester of pregnancy. Lower maternal Vitamin B12 levels when analyzed as a continuous variable was not associated with offspring ADHD (aOR 0.97, 95% CI 0.79–1.18, *p* = 0.75). No significant associations were seen in the lowest quintile of Vitamin B12 levels (aOR 0.96, 95% CI 0.73–1.27, *p* = 0.80). This is the first study examining maternal sera Vitamin B12 levels during early pregnancy and offspring ADHD. The result suggests that Vitamin B12 deficiency during early pregnancy has specificity for some disorders but not with offspring ADHD.

## Introduction

Attention-deficit/hyperactivity disorder (ADHD) is a common neurodevelopmental disorder characterized by symptoms of hyperactivity, impulsivity, and inattention [1]. The estimated prevalence of ADHD is 3.4% in children [2]. Although there is strong evidence for genetic factors in ADHD [1], several environmental factors also contribute [3–7]. Evidence linking maternal nutrition and neuropsychiatric disorders in children has been shown in the Dutch and the Chinese famine studies in which maternal exposure to famine resulted in offspring schizophrenia [8–12] and depression [13, 14]. Maternal nutrition is critical for fetal growth and development [15], and prenatal nutritional deficiency during a critical phase of brain development may result in irreversible functional changes to the brain, predisposing children to neurodevelopmental disorders [16, 17]. Several nutrient deficiencies such as folate, polyunsaturated fatty acids and minerals like iron and iodine in early pregnancy have been reported to negatively impact cognitive and behavioural outcomes of offspring [18–20]. Maternal nutrient deficiency, including folic acid and iron deficits, has been suggested in several birth cohort studies as being related to offspring risk of schizophrenia [21].

Vitamin B12 (Cobalamin) is an essential nutrient required for neural myelination, synaptogenesis and neurotransmitter synthesis [22]. Vitamin B12 functions as an enzyme in the conversion of methylmalonic acid to succinyl-CoA and as a cofactor with folic acid in the methionine synthase reaction, which converts homocysteine into methionine [23]. Impaired Vitamin B12 status during pregnancy has been associated with birth defects [24, 25] and immune function impairment [26]. Folate supplementation has been effective in the prevention of neural tube defects [27, 28].

According to our systematic review on the association of offspring ADHD with maternal Vitamin B12 and folate, we found five relevant studies, summarized in Table 1. None of the studies have examined maternal sera Vitamin B12 and the risk for ADHD in offspring. The UK study including 139 pregnant mothers showed that the maternal red cell folate (RCF) concentration was associated with offspring ADHD symptoms [29]. The Danish register-based study including 35,059 pregnant mothers suggested that multivitamin but not folic acid supplementation before and during early pregnancy was associated with a lower risk of offspring hyperkinetic disorder diagnosis [30]. However, studies from New Zealand (*n* = 6246) [31] and from Spain (*n* = 420) [32] did not find associations between multivitamin or folic acid supplementation during pregnancy and offspring ADHD symptoms The Japanese questionnaire study including 1199 pregnant mothers’ past 1-month diet history found no association between maternal Vitamin B12 intake and parent-rated ADHD symptoms in offspring [33].

The previous studies with inconsistent findings have several limitations. First, no studies have measured Vitamin B12 levels using maternal sera samples and ADHD outcomes in the offspring. Information in previous studies on Vitamin B12 was based on multivitamin supplementation or food intake and the composition of the multivitamins used during pregnancy was not clear. Second, in four of the studies, ADHD symptoms were assessed by parents’ [29, 31, 33] or teachers’ [32] ratings and in only one study, the outcome was derived using register-based diagnoses of ADHD [30]. Third, the doses of supplements were unknown, thus limiting findings and conclusions drawn from these studies.

This is the first population-based study using maternal sera to analyze Vitamin B12 levels in early pregnancy and follow-up of the offsprings’ ADHD outcome. The aim of this study was to examine the association between maternal serum Vitamin B12 levels in early pregnancy and the risk of ADHD diagnosis in the offspring.

## Methods

The study is based on the Finnish Prenatal Study of ADHD (FIPS-ADHD) derived from a nested case–control design. The study includes all singleton live births in Finland from 1998 to 1999 and diagnosed with ADHD in the Care Register for Health Care (CRHC) by 2011. Ethical approval for the study was provided by the Ethics Committee of the Hospital District of Southwest Finland, by the data protection authorities at the National Institute for Health and Welfare, and by the Institutional Review Board of the New York State Psychiatric Institute.

### Nationwide registers

In this study, data were derived from three national registers: the CRHC, the Finnish Medical Birth Register (FMBR), and the Finnish Population Register Center (PRC). These registers are linked using personal identity codes (PIC).

The CRHC includes computerized data on all public and private inpatient diagnoses since 1967 and all outpatient diagnoses since 1998. The diagnostic classification in Finland is based on the International Classification of Diseases (ICD), which is the international standard for reporting diseases and health conditions maintained by the World Health Organization [34]. The ICD-10 has been used since 1996 [34], ICD-9 from 1987 to 1995 [35] and ICD-8 from 1969 to 1986 [36]. A previous diagnostic validation study of the ADHD diagnosis in the register, based on the ICD-criteria for hyperkinetic disorder, has shown 88% validity for the Diagnostic and Statistical Manual of Mental Disorders, Fourth Edition (DSM-IV) diagnostic criteria for ADHD [37]. The ICD-10 and DSM-IV criteria for hyperkinetic disorder/ADHD overlap, but the DSM-IV criteria have been shown to identify a broader group of children [38, 39].

The FMBR includes comprehensive and standardized data on all live births in Finland during the neonatal period up to 7 days of age, and stillbirths where the fetuses had reached at least 22 weeks of gestation or had a birth weight of at least 500 g. Data also include demographic characteristics, reproductive history, maternal health-related behaviors and perinatal events.

The PRC is a computerized national archive that contains basic demographic information on Finnish citizens and permanent residents in Finland. The personal data recorded in the system include name, PIC, address, citizenship and native language, family relations, date of birth and death (if applicable).

### Case and control identification

The Finnish public health care system covers both primary health care and specialized health services and the mental health care services are provided free of charge for both children and adults. Most patients are referred by primary health services, including child welfare clinics and school health care, to specialized services. ADHD is diagnosed by a specialist in psychiatry or neurology in public outpatient services.

The ADHD cases were identified from the CRHC and included all singletons born in Finland between 1998 and 1999 and diagnosed with ADHD based on the ICD-10 codes of hyperkinetic disorders F90.0, F90.1, F90.8, and F90.9 or with the ICD-9 code 314X before 31.12.2011. Cases who had received the diagnosis of severe or profound mental retardation (F72–F73 in ICD-10 or 318 in ICD-9) and who were diagnosed before the age of 2 but not after that were excluded.

The controls were singleton offspring born in Finland and without a diagnosis of ADHD or conduct disorder (F91–F92) or severe or profound mental retardation (F72–73). Each case is matched with one control on date of birth (± 30 days), sex, and place of birth. The controls were alive and residing in Finland at the time of the diagnosis of the matched cases. Among 1672 identified cases and controls, sufficient serum was available in the FMC for 1026 cases and 1026 matched controls.

### Description of biobank in Finland

The Finnish Maternity Cohort (FMC) of the Northern Finland Biobank Borealis [40] was established in 1983 and is a nationwide serum bank with approximately 2 million serum samples collected during the first and early second trimester of pregnancy (5th to 95th percentile: months 2–4 of pregnancy) from over 950,000 women. The FMC covers virtually all pregnancies in Finland during 1983–2016 with archived prenatal serum specimens drawn for routine screening for congenital infections (HIV, Hepatitis B and syphilis). After informed consent, the remaining serum samples (one sample of 1–3 mL for each pregnancy) are stored at − 25 °C in a protected biorepository at the Biobank Borealis and can be used for scientific research [41]. All samples in the FMC can be linked with offspring and other Finnish nationwide registers by a unique PIC that is issued to each resident of Finland since 1971.

### Vitamin B12 measurement

To investigate Vitamin B12 levels in the prenatal serum samples, we measured Active B12 (HoloTC, Holotranscobalamin) using a chemiluminescence microparticle immunoassay (CMIA) on the Architect i2000SR automatic immunoassay analyzer (Abbott Diagnostics) according to the manufacturer’s instructions [42]. The coefficient of variation (mean ± SD) derived from repeated quality control samples included in each set of daily assays was 4.7% in the control samples with high B12 levels (range 43.7–49.6 pmol/L) and 6.4% in those with low B12 levels (range 13.6–17.3 pmol/L).

### Covariates

A number of covariates that have shown to be associated with both Vitamin B12 and ADHD were examined as potential confounders [4, 7, 37, 43–49]. We obtained information on the number of previous births, maternal socioeconomic status (SES), maternal age, maternal smoking during pregnancy, gestational age and weight for gestational age from the FMBR. In Finland, the maternal SES categories are based on occupation-based classification that is used in the FMBR: upper white-collar workers, lower white-collar workers, blue-collar workers and others (e.g. students and housewives) or missing (if the data were not available). Information on maternal and paternal psychiatric diagnoses and maternal substance abuse was obtained from the CRHC [4]. Information on maternal immigrant status was obtained from the PRC and included in the analysis, as maternal nutrition deficiency is more common in certain ethnic groups [50] and there is an increased risk for offspring ADHD diagnosis [45]. The FMC was used for information on gestational week and season of blood draw. The seasons of blood collection were defined as winter (December to February), spring (March to May), summer (June to August) and fall (September to November). More description of all covariates is presented in Tables 2 and 3.

### Statistical analysis

We examined the association between Vitamin B12 levels as a continuous variable and ADHD. Due to the skewed distribution, Vitamin B12 was log-transformed before analysis. In the secondary analysis, we examined maternal Vitamin B12 classified as quintiles. The cut-off of the quintiles for case and control groups was based on the distribution in the control group. The reference group was the highest quintile. The categorically defined covariates were tested with *χ*^2^ and Student’s *t* tests for the association with log-transformed maternal Vitamin B12 levels among controls, and for the association with ADHD. The covariates were included in the adjusted regression models as potential confounders if they were associated with both the exposure and the outcome at *p* < 0.1. The point and interval estimate of odds ratios were obtained by fitting conditional logistic regression models for matched pairs. Statistical significance was based on *p* < 0.05. All the statistical analyses were performed with (SAS 9.4, SAS Institute, Cary, N.C.)

## Results

The study included 1026 ADHD case–control pairs. The mean age of diagnosis for cases was 7.3 years (SD 1.9, range 2–14 years).

Table 2 shows the association between covariates and maternal Vitamin B12 among controls. Gestational week of blood draw and maternal SES were associated with maternal Vitamin B12 levels among controls. Table 3 shows the association between covariates and offspring ADHD among case and control subjects. Maternal age, previous births, history of maternal ADHD diagnoses, maternal substance abuse, maternal psychopathology, paternal ADHD diagnoses, paternal psychopathology, maternal smoking, maternal SES, gestational age, weight for gestational age and maternal immigration status were associated with offspring ADHD. Therefore, adjustment was made only for maternal SES.

Table 4 shows the unadjusted and adjusted results for the association between maternal serum Vitamin B12 and offspring ADHD. The maternal Vitamin B12 levels, analyzed as a continuous variable, were not associated with offspring ADHD in either the unadjusted (OR 0.96, 95% CI 0.79–1.16, *p* = 0.68) or the adjusted analyses (aOR 0.97, 95% CI 0.79–1.18, *p* = 0.75). Maternal Vitamin B12 levels, measured in quintiles, were also not associated with offspring ADHD. There was no significant association between the lowest quintile of maternal Vitamin B12 and ADHD in either the unadjusted (OR 0.96, 95% CI 0.73–1.26, *p* = 0.77) or the adjusted analyses (aOR 0.96, 95% CI 0.73–1.27, *p* = 0.80). When an additional analysis was conducted adjusting for all covariates for maternal Vitamin B12 levels measured as a continuous variable, and in quintiles, the findings did not change. As ADHD diagnoses may be considered unreliable before the age of 6 years, we conducted sensitivity analyses, including cases diagnosed at age 6 years or more (*n* = 948). These cases might have been diagnosed before age 6, but they had to have the diagnosis recorded at six years or more. The findings remained negative in all analyses (Supplementary Table 1).

## Discussion

This is the first population-based study examining maternal Vitamin B12 levels in prenatal sera in relation to ADHD in offspring. There was no significant association observed between maternal Vitamin B12 and offspring ADHD. The findings of the study were in line with previous studies based on folate and multivitamin supplementation [31–33]. In contrast, the only previous study with offspring ADHD diagnoses from Denmark showed that multivitamin supplementation was associated with a reduced risk for ADHD in offspring [30]. However, the Danish study did not include the levels of serum Vitamin B12 during pregnancy.

The major strengths of the present study are the large nationwide representative sample and availability of maternal Vitamin B12 levels from prospectively collected maternal serum samples during pregnancy. The study was well powered to detect even a relatively small difference between case and control levels of maternal Vitamin B12. Assuming two independent samples and given the number of cases and controls (*n* = 1026 per group) with alpha set at 5% for a two-sided *t* test, at 80% power the minimum detectable effect size would have been *d* = 0.12 (in units of SD), generally considered a “small” effect size.

We note some limitations to our study. First, ADHD is a multifactorial disorder involving a combination of genetic and environmental factors; yet, the present study concerns only Vitamin B12, a single nutrient. However, as in most research studies of complex outcomes, effects can still be observed for single risk factors including micronutrients as in studies of folic acid and neural tube defects [21, 51], and the reduction in risk obtained is also of public health benefit even though the outcome is not entirely eliminated by this intervention. The ultimate goal of this research is to improve the explanatory variance of ADHD, and for that purpose, other groups and we are working to increase the number of micronutrients and other factors quantified. With regard to the present study, folic acid and homocysteine are not measurable in the stored sera; however, one advantage of quantifying serum levels is that it represents a final outcome of such factors, including genes related to homeostatic and metabolic processes and, as in the folic acid/neural tube defects findings, the pathology can be corrected by supplementation of this nutrient even without directly addressing the antecedent factors [21]. Second, we did not quantify neonatal levels of Vitamin B12. However, maternal and neonatal serum levels of Vitamin B12 were significantly correlated with one another in prior work [51]. Third, ADHD cases were referred to specialized services and are likely to represent more severe cases. Fourth, ADHD diagnoses were based on register information. However, our previous study has reported the validity of ADHD diagnosis to be high (88%) [37]. Fifth, information about serum Vitamin B12 levels was restricted to the first and early second trimesters of pregnancy, and thus the findings cannot be generalized to those occurring later in pregnancy.

The present study does not support the hypothesis that Vitamin B12 deficiency during pregnancy is an etiological factor for offspring ADHD. Some previous studies have suggested that maternal multivitamin or folate supplementation and high homocysteine levels might have an association with offspring neuropsychiatric disorders such as autism and schizophrenia [52–55]. In a large birth cohort, the Child Health and Development Study, Brown et al. [55] demonstrated that maternal homocysteine levels in the highest tertile of the distribution were related to a greater than twofold increased risk of schizophrenia among offspring. It has been suggested that one possible explanation for these associations could be related to dysfunction in one-carbon metabolism resulting in altered DNA methylation [56]. Low folate causes high homocysteine in maternal serum through a disruption in methionine metabolism, which may induce abnormal fetal brain development. Of note, the present study is not comparable with previous studies on other neuropsychiatric disorders that have not specifically measured Vitamin B12 levels during pregnancy. Future studies should address the possibility that maternal Vitamin B12 deficiency has specificity as an etiological factor for some disorders but not others as well as the possibility that Vitamin B12 could be related to offspring ADHD only in mothers or offspring with a genetic mutation that alters one-carbon metabolism. This has been suggested in studies of gene variants such as methylene tetrahydrofolate reductase (MTHFR), folic acid, and neural tube defects [57].

## Figures and Tables

**Table 1:** Literature review of maternal Vitamin B12, folate and/or multivitamin supplement and ADHD

S. no.	Author/Country/Title	Diagnosis	Study design	Diagnostic criteria/data source	Period of supplementation	Dosage	Sample size/Age	Covariates	Results
1	D’Souza et al. [31] (New Zealand)	ADHD symptoms	Prospective cohort (growing up in New Zealand)	Hyperactivity-inattention symptoms assessed by mother-report SDQ	Folic acid and multivitamin supplementation before pregnancy, during the first trimester, and after the first trimester assessed in late pregnancy	Not available	6246/2 years	Mother’s ethnicity, mother’s education, mother’s age when pregnant, child’s gender, child’s gestational age, child’s birth weight, parity, planned pregnancy, mother in paid employment, area-level deprivation, and rurality	Multivitamin was not associated with hyperactivity-inattention symptoms (OR 0.97, 95% CI 0.75–1.24) Compared to pre-conception and 1^st^ trimester folic intake, both 1^st^ trimester only (OR 0.98, 95% CI 0.74–1.31) and no intake (OR 0.88, 95% CI 0.57–1.34) were not associated with hyperactivity-inattention symptoms
2	Miyake et al. [33] (Japan)	ADHD symptoms	Prospective cohort Kyushu Okinawa Maternal and Child Health Study (KOMCHS)	Hyperactivity-inattention symptoms assessed by parent-report SDQ at age 5 years	Folate and other B-vitamin intake from food in the past month assessed by Diet history questionnaire (DHQ) at 5–39 weeks (no information of supplementation recorded)	Average consumption of food and beverages^1^	1199 3–16 years	Maternal age, gestation at baseline, region of residence, number of children, maternal and paternal education, household income, maternal depressive symptoms during pregnancy, maternal alcohol intake during pregnancy, maternal Vitamin B complex supplement use during pregnancy, maternal smoking during pregnancy, child’s birth weight, child’s sex, breastfeeding duration, and smoking in the household during the first year of life	Vitamin B12 from food was not associated with hyperactivity-inattention problems, quartiles Q2 (OR 0.80, 95% CI 0.49–1.29), Q3 (OR 0.99, 95% CI 0.61–1.61) and Q4 (OR 0.81, 95% CI 0.50–1.32) Folate from food was not associated with hyperactivity-inattention problem, quartiles Q2 (OR 0.75, 95% CI 0.46–1.21), Q3 (OR 0.66, 95% CI 0.40–1.07) and Q4 (0.69, 95% CI 0.42–1.12)
3	Virk et al. [30] Denmark	Hyperkinetic disorder	Prospective cohort (Danish National Birth Cohort (DNBC)	ICD-10 HKD diagnosis (F90.0–F90.9) at or after their fifth birthday, ADHD symptoms assessed by Parent-report SDQ for children at age 7 years	Folic acid and multivitamin supplementation from (4–8 weeks) assessed at 12 weeks	Not available	35059 Age:7 years	Maternal age, household SES, maternal smoking, alcohol consumption during pregnancy, maternal pre- pregnancy BMI, birth year, offspring’s sex	Multivitamin intake was associated with lower risk for HKD diagnosis (aHR 0.70, 95% CI 0.52–0.96) and ADHD medication (aHR: 0.79, 95% CI 0.62–0.98) Folic acid intake was not associated with risk for HKD diagnosis (aHR 0.87, 95% CI 0.54–1.41) Multivitamin intake was not associated with parent report hyperac-tivity-inatten-tion symptoms (aRR 0.90, 95% CI 0.76–1.06) however folic acid intake was associated with parent report hyperactivity-inattention symptoms (aRR 0.62, 95% CI 0.47–0.94)
4	Schlotz et al. [29] UK	ADHD symptoms	Prospective cohort	Mother-report SDQ at age 8 years	Maternal red blood cell folate (RCF), measured at 14 weeks Total folate intake (TFI) from food and supplement assessed in early and late pregnancy assessed by Food frequency questionnaire (FFQ) at 14 weeks and during late pregnancy at 28 weeks	Frequency of consumption of 100 foods or food groups^2^	139 Age:7.6–9.8 years	Sex, mother’s tobacco or alcohol consumption during pregnancy, education, daily energy intake	Lower maternal red cell folate concentration (RCF: beta β = −0.24, 95% CI − 2.20, −0.26) and total folate intake from food and supplements (TFI: beta β = −0.24, 95% CI − 1.39, −0.11) in early pregnancy were both associated with higher hyperactivity-inattention symptoms However, total folate intake from food and supplements in late pregnancy (TFI: beta β = 0.02, 95% CI − 0.80, 0.93) was not associated with hyperactivity-inattention symptoms
5	Julez et al. [32] Spain	ADHD symptoms	Prospective birth cohort from Menorca	ADHD-DSM-IV criteria-Psychologists and Teacher rated Cut-off 80^th^ percentile	Folic acid and multivitamin supplementation assessed at the end of first trimester of pregnancy	Not available	420 children Age: 4 years	Parental social class and level of education, mother’s parity at child’s age four, mother’s marital status, tobacco smoking during pregnancy, maternal intake of supplementary calcium and iron at same time as study determinants, gestational age at interview, child’s gender, child’s duration of breast feeding, child’s age and school season during test assessment, evaluator (psychologist), child’s home location at age four	Vitamin use without folic acid was not associated with ADHD (OR 0.26, 95% CI 0.05–1.31); Hyperactivity symptoms (OR 1.07, 95% CI 0.26–4.45) and Inattention symptoms (OR 0.24, 95% CI 0.05–1.33) Folic acid with or without other Vitamins was not associated with ADHD (OR 0.74, 95% CI 0.38–1.47). Hyperactivity symptoms (OR 1.44, 95% CI 0.6–3.03) but associated with inattention symptoms (OR 0.46, 95% CI 0.22–0.95)

Abbreviations: ADHD, Attention-deficit hyperactivity disorder; ; SDQ, Strength and Difficulty Questionnaire; OR, Odd’s ratio; CI, Confidence Interval; KOMCHS, Kyushu Okinawa Maternal and Child Health Study; DHQ, Diet history questionnaire; ; DNBC, Danish National Birth Cohort; HKD, Hyperkinetic disorder; aHR, Adjusted Hazard Ratio; RCF, Red Blood Cell Folate; TFI, Total folate intake; FFQ, Food frequency questionnaire; DSM-IV, Diagnostic and Statistical Manual of Mental Disorders; BMI, Body Mass Index; UK, United Kingdom

1 Average consumption of eight categories of food ranging from ’never’ to ≥ 2 times/d” for foods and from “< 1 time/wk” to “≥ 6 times/d” for beverages. The relative portion size was compared with a standard portion size according to five categories: “50% smaller or less,” “20%–30% smaller,” “same,” “20%–30%larger,” and “50% larger or more”.

2 Average frequency of consumption of 100 foods or food groups in the preceding three months. The nutrient content of a standard portion of each food was multiplied by its reported frequency of use and supplement use was ascertained in detail, allowing calculation of average total intakes of energy (kcal/day) and folate (TFI; μg/day).

**Table 2:** Relationship between covariates and maternal Vitamin B12 levels (≥/< median) among controls

Covariates	Maternal Vitamin B12		Maternal Vitamin B12			P-value
	≥ Median		< Median			
	Mean	SD	Mean	SD	t	
**Maternal age (years)**	29.52	5.20	29.58	5.41	0.18	0.85
**Gestational week of blood draw**	10.19	2.89	11.06	3.21	4.58	<**0.001**
	N	%	N	%	*χ* ^2^	
**Previous births**						
0	191	37.23	209	40.74	1.32	0.25
≥1	322	62.77	304	59.26		
**History of maternal ADHD diagnosis** ^a^					1.00	0.50
No	512	99.81	513	100		
Yes	1	0.19	0	0		
**History of maternal substance abuse** ^b^					0	1.00
No	506	98.64	506	98.64		
Yes	7	1.36	7	1.36		
**History of maternal psychopathology** ^c^					0.452	0.50
No	455	88.69	448	87.33		
Yes	58	11.31	65	12.67		
**History of paternal ADHD diagnosis**^a^*^1^					1.0	0.49
No	511	100	507	99.80		
Yes	0	0	1	0.20		
**History of paternal psychopathology**^d^*^1^					0.003	0.95
No	443	86.69	441	86.81		
Yes	68	13.31	67	13.19		
**Maternal smoking** ^2^					0.12	0.72
No	432	86.57	434	87.32		
Yes	67	13.43	63	12.68		
**Maternal SES**					10.24	**0.03**
Upper white collar	71	13.84	67	13.06		
Lower white collar	213	41.52	234	45.61		
Blue collar	106	20.66	77	15.01		
Others	68	13.26	91	17.74		
Missing	55	10.72	44	8.58		
**Gestational age (weeks)** ^**3**^					0.24	0.62
<37	19	3.71	22	4.31		
≥37	493	96.29	488	95.69		
**Weight for gestational age** ^**3**^					1.33	0.51
<−2 SD	10	1.95	13	2.55		
−2 SD to +2 SD	480	93.75	481	94.31		
>+2 SD	22	4.30	16	3.14		
**Maternal immigration status**					0.10	0.28
No	**511**	99.61	507	98.83		
Yes	2	0.39	6	1.17		

a ICD-10: F90.X or ICD-9: 314.X,

b ICD-8 (291, 303, 304), ICD-9 (291, 292, 303,304,305) or ICD-10 (F10–19);

c ICD-8 (291–308), ICD-9 (291–316) or ICD-10 (F10–99, excluding mental retardation (F70–79) excluding maternal substance abuse diagnosis,

d ICD-8 (291–308), ICD-9 (291–316) or ICD-10 (F10–99, excluding mental retardation (F70–79).

1 Data missing for 7 controls;

2 Data missing for 30 controls; Data missing for 4 controls; SD: standard deviation; t: t-test value; *χ*^2^: Pearson’s chi squared test value.

**Table 3:** Relationship between covariates and ADHD in case and control subjects

Covariates	Cases		Controls			P-value
	Mean	SD	Mean	SD	t	
**Maternal age (years)**	27.89	5.87	29.55	5.31	6.73	<**0.001**
**Gestational week of blood draw**	10.72	3.64	10.63	3.08	−0.61	0.54
	N	%	N	%	*χ* ^2^	
**Previous births**					14.69	< **0.001**
0	486	47.37	400	38.99		
≥ 1	540	52.63	626	61.01		
**History of maternal ADHD diagnosis** ^a^					4.51	0.03
No	1019	99.32	1025	99.90		
Yes	7	0.68	1	0.10		
**History of maternal substance abuse** ^b^					26.09	**<0.001**
No	970	94.54	1012	98.64		
Yes	56	5.46	14	1.36		
**History of maternal psychopathology** ^c^					60.25	**<0.001**
No	766	74.66	903	88.01		
Yes	260	25.34	123	11.99		
**History of paternal ADHD diagnosis**^a^*					6.58	**0.01**
No	991	99.10	1018	99.90		
Yes	9	0.90	1	0.10		
**History of paternal psychopathology**^d^*					41.82	**<0.001**
No	755	75.50	884	86.75		
Yes	245	24.50	135	13.25		
**Maternal smoking** ^**1**^					83.31	**<0.001**
No	701	70.17	866	86.95		
Yes	298	29.83	130	13.05		
**Maternal SES**					35.19	**<0.001**
Upper white collar	71	6.92	138	13.45		
Lower white collar	408	39.77	447	43.57		
Blue collar	214	20.86	183	17.84		
Others	205	19.98	159	15.50		
Missing	128	12.48	99	9.65		
**Gestational age (weeks)**					25.07	**<0.001**
<37	98	9.59	41	4.01		
≥37	924	90.41	981	95.99		
**Weight for gestational age**					11.14	**0.003**
<−2 SD	51	5.00	23	2.25		
−2 SD to +2 SD	936	91.67	961	94.03		
>+2 SD	34	3.33	38	3.72		
**Maternal immigration status**					6.63	**0.01**
No	1004	97.86	1018	99.22		
Yes	22	2.14	8	0.78		

a ICD-10: F90.X or ICD-9: 314.X,

b ICD-8 (291, 303, 304), ICD-9 (291, 292, 303,304,305) or ICD-10 (F10–19);

c ICD-8 (291–308), ICD-9 (291–316) or ICD-10 (F10–99, excluding mental retardation (F70–79) excluding maternal substance abuse diagnosis,

d ICD-8 (291–308), ICD-9 (291–316) or ICD-10 (F10–99, excluding mental retardation (F70–79); SD: standard deviation; t: t-test value; χ^2^: Pearson’s chi squared test value.

**Table 4:** Odds ratios and 95% CI of the association between maternal serum Vitamin B12 (continuous, quintiles) and offspring ADHD

A. Vitamin B12 as a continuous variable						
	Case (N=1026)	Control (N=1026)	Association with maternal serum Vitamin B12			
Maternal Vitamin B12 levels (pmol/L)	Median	Median	Odds ratio (unadjusted) 95% CI	P-value	Odds ratio (adjusted*) 95% CI	P-value
Log-transformed analysis	4.50	4.52	0.96 (0.79–1.16)	0.685	0.97 (0.79–1.18)	0.759
B. As a categorical variable						
	Case (N=1026)	Control (N=1026)	Association with maternal serum Vitamin B12			
Maternal Vitamin B12 (%) (pmol/L)	n (%)	n (%)	Odds ratio (unadjusted) 95% CI	P-value	Odds ratio (adjusted*) 95% CI	P-value
**Quintiles**						
< 20 (< 1.20)	202 (19.68)	202 (19.68)	0.96 (0.73–1.26)	0.771	0.96 (0.73–1.27)	0.803
20–39 (1.20–2.38)	219 (21.34)	205 (19.98)	1.02 (0.77–1.34)	0.883	1.03 (0.78–1.37)	0.802
40–59 (2.39–4.02)	206 (20.07)	205 (19.98)	0.95 (0.72–1.26)	0.758	0.98 (0.74–1.29)	0.892
60–79 (4.03–7.09)	184 (17.93)	208 (20.27)	0.84 (0.63–1.11)	0.236	0.87 (0.65–1.16)	0.360
≥80 (≥7.10)	215 (20.95)	206 (20.07)	Reference		Reference	

* Adjusted for maternal SES
